# Combined Surgically Induced Macular Detachment and Autologous Internal Limiting Membrane Transplantation for Refractory Full Thickness Macular Hole

**DOI:** 10.3390/jcm14062123

**Published:** 2025-03-20

**Authors:** Rino Frisina, Laura Di Leo, Ilenia Gallo Afflitto, Andrea Vulpetti, Lorenzo Motta, Gabriella De Salvo

**Affiliations:** 1 Ophthalmology Unit, Surgery Department, Guglielmo da Saliceto Hospital, 29121 Piacenza, Italy; l.dileo@ausl.pc.it (L.D.L.); i.galloafflitto@ausl.pc.it (I.G.A.); a.vulpetti@ausl.pc.it (A.V.); 2 Department of Ophthalmology, School of Medicine, University of Padova, 35121 Padova, Italy; drlorenzomotta@gmail.com; 3 Department of Ophthalmology, University Hospital Southampton NHS Foundation Trust, Southampton SO16 6YD, UK; gabrielladesalvo@gmail.com; 4 Faculty of Medicine, University of Southampton, Southampton SO17 1BJ, UK

**Keywords:** full thickness macular hole, autologous internal limiting membrane, best corrected visual acuity, vitrectomy, macular detachment

## Abstract

**Background/Objectives**: To propose a combined surgery of surgically induced macular detachment (MD) and autologous internal limiting membrane (ILM) transplantation to treat refractory full thickness macular holes (FTMHs). **Methods**: A series of patients affected by refractory FTMHs underwent a combined surgery. The following demographic and clinical data were collected: age, gender, eye, lens status, and best corrected visual acuity (BCVA). The tomographic pre- and post-operative parameters were the following: pre-operative FTMH diameter, refractory FTMH morphology (flat/with cuff), FTMH closure, foveal profile (regular/flat/inverted), flap displacement, and outer retinal layers restoration. **Results:** The study included a total of 14 pseudophakic eyes (14 patients). In all of the patients, surgical FTMH closure was reached. The mean BCVA improved after surgery from 1.1 ± 0.14 to 0.48 ± 03 logMAR (*p* < 0.0001). Statistical analysis demonstrated that the larger the FTMH, the poorer the post-operative gain in BCVA (*p* −0.5). The post-operative regular foveal profile was obtained in 50% of the eyes with a mean post-operative BCVA of 0.3 logMAR. A negative correlation between the time interval from diagnosis to surgery and post-operative BCVA gain was highlighted (*p* −0.8). **Conclusions**: The proposed combined surgical technique led to encouraging anatomical and functional results. Surgically induced MD increased the elasticity of the retina, and the free flap isolated the macular hole from the vitreous chamber favoring its closure.

## 1. Introduction

Primary full thickness macular hole (FTMH) closure rates after pars plana vitrectomy (PPV) combined with the peeling of the internal limiting membrane (ILM) is reported to range from 84% and 94% [1,2,3,4,5,6,7]. Despite the high closure rates, there remains a subset of cases in which the hole fails to close. This failure can be attributed to a multitude of factors that have been extensively discussed in the literature [8]. Among those, intrinsic factors related to the history and morphology of FTMHs are of paramount importance. For instance, large macular holes, typically those with a minimum diameter exceeding 400 μm, are known to be more difficult to close [9,10,11]. Similarly, chronic FTMHs can lead to progressive degenerative changes within the retinal tissue, further compromising the potential to obtain a successful anatomical restoration. Such degenerative changes often manifest as tissue thinning or scarring, which in turn impede the retinal natural healing process [12,13,14]. Furthermore, the success of a macular hole closure is heavily influenced by technical aspects of the surgical procedure. In fact, an incomplete or inaccurate vitrectomy, which results in the persistence of residual vitreous and the associated tractional forces, can significantly hinder the closure process. Incomplete separation of the posterior vitreous is another technical shortcoming that may contribute to the failure of the adequate closure of the hole. Moreover, variables such as the extent and completeness of the ILM peeling have been shown to play a crucial role in determining the outcome of the procedure [8,10,14]. A less extensive or inconsistent peeling may leave behind residual cortical vitreous or cellular membranes that continue to exert tangential traction on the retina [15,16,17]. In addition, patient-specific factors such as age need also to be taken into account: older patients may have a lower capacity for tissue recovery, negatively impacting the hole closure. Finally, a thin residual retinal thickness around the hole is typically indicative of a significant loss of neural tissue, which further decreases the likelihood of a complete closure being achieved [8,9,10,11,12,13,14].

Refractory FTMH is defined as an unclosed or reopened FTMH after standard surgery, pars plana vitrectomy (PPV), and ILM peeling. Managing these cases remains a significant surgical challenge. Failure to achieve the closure can lead to progressive visual deterioration and a decline in the patient’s quality of life. The progressive visual loss only affects daily activities but may also have profound psychological and social implications for affected individuals.

Over the years, the quest to improve the anatomical and functional outcomes in cases of refractory FTMHs has led to the development of a variety of alternative surgical techniques. One of the earliest approaches involved the use of permanent tamponades, such as silicone oil (SO), either alone or in combination with blood derivates, to enhance the process of macular hole closure [18]. However, these approaches do not always ensure satisfactory anatomical and functional outcomes. These limitations have spurred further research into more innovative strategies.

More recently, several novel surgical techniques have emerged based on innovative principles, including ILM flap manipulation, autologous retinal grafting (ARG), an autologous retinal transplant (ART), human amniotic membrane grafting (hAMG), a multilayer ILM plug (MIP), and chorioretinal adhesives [18,19,20,21,22,23,24]. The unifying concept underlying these techniques is the use of an additional tissue or biomaterial to serve as a scaffold, which in turn stimulates cellular regeneration and supports the reestablishment of retinal continuity. This scaffold-based approach is designed to encourage the proliferation of reparative cells and to promote the ingrowth of supportive glial tissue, thereby facilitating the closure of the macular hole and the restoration of retinal architecture [10,11,12,18].

A particularly innovative approach was introduced by Oliver et al. in 2011 [25]; their proposal diverged from traditional techniques through their focus on the induction of macular detachment (MD) by injecting a balanced saline solution (BSS) with a 41-gauge needle into the subretinal space. This technique aims to reduce the tension of the retina increasing its elasticity by detaching the neuroepithelium from the retinal pigment epithelium (RPE) in the macula area and inducing FTMH closure with the use of a subsequent gas tamponade [25]. This surgical technique does not affect the possibility of possible combined surgical procedures. More specifically, the use of a free ILM flap that acts as a scaffold by inducing the proliferation and gliosis of Muller cells, in combination with the surgically induced MD technique may increase the surgical success rate, i.e., FTMH closure.

In the present manuscript, the authors present the results of their audit assessing the results of the aforementioned combined surgical technique, which integrates the induction of a macular detachment with an autologous ILM transplantation for the treatment of refractory FTMHs. This combined approach offers a twofold advantage: the increase of the retinal elasticity, and the supply of a biological scaffold that triggers cellular regeneration and proliferation. The aim of this study is to evaluate whether this combined surgical strategy produces satisfactory anatomical and functional outcomes in the repair of refractory FTMHs. All procedures and protocols described in the study have been conducted in strict adherence to the principles outlined in the Declaration of Helsinki, ensuring the highest ethical standards in clinical research were maintained. Through this comprehensive evaluation, the authors aim to provide insights into the potential benefits and limitations of combining surgically induced macular detachment with autologous ILM transplantation. Detailed analysis of this technique may pave the way for improved management of refractory macular holes, ultimately leading to better visual outcomes and an enhanced quality of life for patients facing this challenging condition.

## 2. Materials and Methods

### 2.1. Design: Interventional Case Series

**Figure 1:**
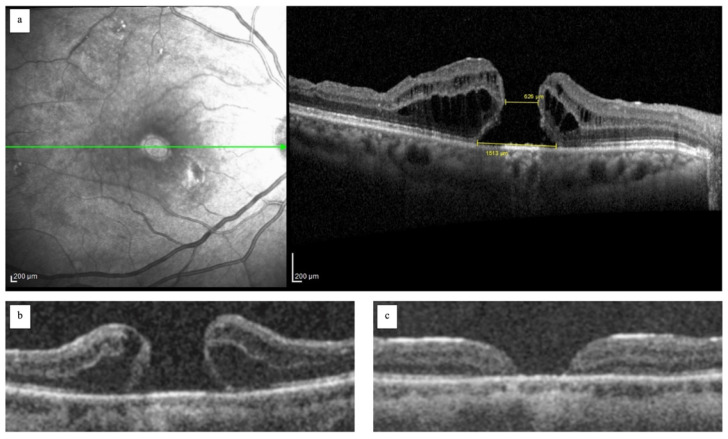
Image (**a**) shows the measurements of the base diameter and minimum diameter in a refractory full thickness macular hole (FTMH); image (**b**) shows a refractory FTMH with a cuff; image (**c**) a flat refractory FTMH.

A series of consecutive patients affected by refractory FTMHs after PPVs and ILM peeling were recruited at the Ophthalmology Unit of Guglielmo da Saliceto Hospital, Piacenza (Italy). All patients underwent a combined surgically induced MD and autologous ILM transplantation, gas tamponade (20% Sulfur Hexafluoride, SF6), and pos-toperative face-down position. This study was approved by the local ethics committee. At the time of surgery, every patient was informed about the aims, methods, benefits, and potential risks of the treatment. Informed consent was obtained from all patients. The following demographic and clinical data were collected: age (years), gender (male/female), eye (right/left), lens status (phakic, pseudophakic), best corrected visual acuity (BCVA) reported with the Snellen chart (SC) and a logarithm of the minimum angle of resolution scale (logMAR). High-resolution spectral-domain optical coherence tomography (SD-OCT, Spectralis, Heidelberg Engineering, Heidelberg, Germany) was performed to evaluate the following morphological parameters: the pre-operative base diameter and minimum diameter of FTMH measured in μm, and the morphology of FTMHs (i.e., flat or with cuff) (Figure 1). As for the diameters of the macular hole, horizontal and vertical linear tomographic scans were performed centered on the macular hole. The base diameter was the distance, along the RPE profile, between the point where the retinal tissue breaks and the RPE is exposed and the same point on the opposite side. The minimum diameter was considered the shortest distance between the edges of the macular hole chosen between the diameters of the macular hole from the base to the apex of macular hole (Figure 1). Concerning the morphology of the hole, FTMHs with cuffs were defined by when the edges of the FTMH were detached and elevated; flat FTMHs were defined by when the edges of the hole were adherent to the RPE (Figure 1).

**Figure 2:**
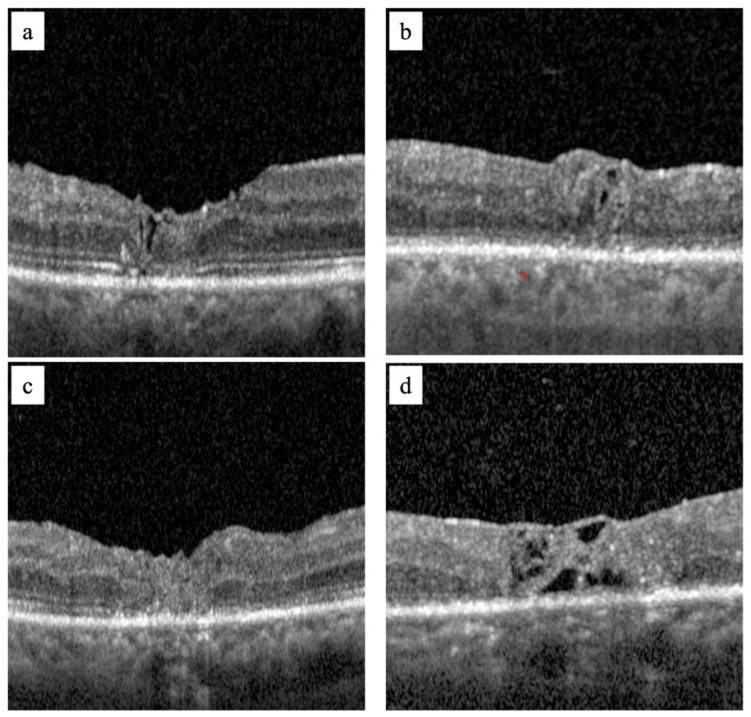
Image (**a**) shows a refractory FTMH closure with a regular foveal pattern and almost complete outer retinal layers (ORL) restoration; image (**b**) shows a refractory FTMH closure with an inverted foveal profile pattern with complete ORL restoration; image (**c**) shows a refractory FTMH closure with a flat foveal pattern and foveal atrophy: image (**d**) shows a refractory FTMH closure with foveal atrophy and partial integration of the flap into the foveal tissue.

At 6 months, post-operative BCVA and the following tomographic parameters were analyzed: FTMH closure, foveal profile (regular, flat, or inverted), flap displacement, and restoration of outer retinal layers (ORL) (Figure 2). As regards the foveal profile, the authors defined the foveal profile as regular when the foveal depression was detectable, flat when the foveal depression was absent, inverted when the flap inside the hole exceeded in volume such as to determine a convex foveal profile (Figure 2).

Pre- and post-operative morphological tomographic parameters and surgical time were correlated with post-operative BCVA in order to evaluate whether there are prognostic factors indicative of functional recovery.

### 2.2. Surgical Technique

All surgeries were performed by the same senior vitreoretinal surgeon (RF). To induce an MD, we used a 41-gauge needle connected to the injection line of the vitrectomy machine (25-gauge system, Stellaris Elite™ PC Vision Enhancement System, Bausch & Lomb, Bridgewater, NJ, USA). The infusion was controlled by a foot pedal with a pressure range of 0 to 30 mmHg. Three retinotomy punctures were made. An MD was completely achieved by performing several fluid–air exchanges. The air, filling the vitreous chamber, pushed the subretinal fluid, conveying it to the posterior pole, inducing a complete MD. Once a total MD was obtained with the edges of the FTMH raised, blue dye was injected under air and then perfluorocarbon liquid (PFCL). Afterwards, an air–fluid exchange was performed. A free ILM flap was peeled off and inserted into the FTMH. PFCL was removed and a final 20% SF6 tamponade was performed. The surgical video shows the steps of the combined surgery (Video S1).

### 2.3. Statistical Analysis

Absolute and relative frequencies were used to describe the following qualitative parameters: gender, eye, lens status, and morphology of the FTMH. Quantitative parameters—such as age, AL, BCVA, base diameter, and minimum diameter—were described using a mean value, standard deviation, and range. Quantitative variables were compared by means of the Student’s *t*-test, while qualitative ones were compared by means of the χ^2^ test. The SPSS software version 22.0 (IBM Corporation, New York, NY, USA) was used to process statistical analysis.

## 3. Results

A total of 14 consecutive pseudophakic eyes (6 right) from 14 patients (6 females) affected by refractory FTMHs who underwent a combined surgery of surgically induced MD and free ILM flap techniques were enrolled. The demographic and pre-operative clinical data are reported in Table 1. The closure of the FTMH was reached in all eyes. The mean BCVA improved after surgery from 1.1 ± 0.14 logMAR (range 1.4–1), to 0.48 ± 0.3 logMAR (range 1.1–0.3) (*p* < 0.0001). Statistical analysis of the correlation between the mean of FTMH diameters (base and minimum diameters) and the post-operative gain of BCVA demonstrated that the larger the FTMH, the poorer was the post-operative gain of BCVA (Pearson correlation −0.4). The post-operative regular foveal profile was obtained in 50% of eyes with a mean post-operative BCVA of 0.3 logMAR. The eyes with flat (5 eyes) and inverted foveal profiles (2 eyes) had a mean post-operative BCVA of 0.6 logMAR with no statistically significant differences observed. A negative correlation between the time interval from diagnosis to surgery and post-operative BCVA gain was also highlighted (Pearson correlation −0.8).

## 4. Discussion

**Figure 3:**
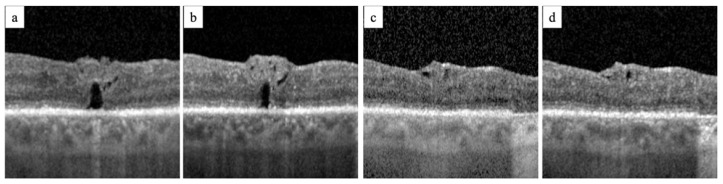
The tomographic morphological changes to a FTMH after combined surgery, surgically induced macular detachment and autologous internal limiting membrane transplantation. In image (**a**), an OCT scan of the FTMH shows the free ILM flap covering FTMH creating a subfoveal space isolated from the vitreous chamber. Image (**b**) shows the reduction of the subfoveal space with the approaching of the edges of the FTMH. In images (**c**,**d**) the FTMH has completely closed with the disappearance of the subfoveal space and the restoration of the outer retinal layers.

The rationale for combined surgery is that the hole-closing mechanisms that each surgical techniques alone can induce may synergically increase the successful rate of FTMH closure and potentially allow for a complete foveal restoration. The standard surgical management for primary FTMHs consists of ILM peeling to free the retina from the centripetal forces that caused FTMH development [26,27,28]. Refractory FTMH is the result of residual tractional forces or of persistent retinal stiffness; the surgically induced MD allows for an increase in the elasticity of the retina [26,27,28]. In our experience, the mechanism behind FTMH closure with the restoration of intraretinal layers and with a potential functional recovery occurs with a temporal sequence of events. The first event is the correct placement of the ILM flap in contact with the edges of the FTMH at the level of the inner retinal layers (IRL), covering the FTMH without filling it completely by encountering the bared RPE. This creates a pre-epithelial sub-foveal space and the RPE is covered and isolated from the vitreous chamber. Figure 3a shows the free ILM flap placed on the retinal surface in contact with the IRL, covering the FTMH without filling it and creating an isolated pre-epithelial sub-foveal space. Subsequently, the sub-foveal space (Figure 3b–d) reduces in volume until the FTMH is completely closed and all retinal layers are restored. Our hypothesis is that covering the FTMH and isolating it from the vitreous chamber creates a closed environment that allows for the stimulation of gliosis in Muller cells due to the release of neurotrophic factors, bFGF, and humoral factors. Studies on macular holes have found that migration and Muller cell gliosis are induced in dry environments [26,27,28].

The aim of surgery is to induce cellular gliosis to obtain the closure of the macular hole. However, the complete restoration of the retinal layers and visual function can be compromised by the same repair events. Glial repair is not controllable, and its outcomes can vary. It may lead to retinal tissue repair, restoring the integrity of the retinal layers and allowing for the recovery of visual function. This process does not always occur in such a sequential manner, and foveal atrophy may indeed occur due to an excessive fibrotic reaction that causes a permanent loss of central visual function [26,27,28]. The main limits of autologous ILM transplantation are the inability to predict how the free ILM flap positions itself in the FTMH and to control gliosis by the Müller cells. We believe, based on our experience, that both limits are strongly influencing each other. Several authors have attempted to describe how the free flap fits inside the FTMH [18,29,30,31], but it probably depends on several factors, not all of which are understood. Nevertheless, based on the evidence, if the flap fills the FTMH entirely, it may itself represent an impediment to cell re-proliferation rather than a scaffold. Figure 2c shows an OCT scan of the post-operative closure of an FTMH that highlights a FTMH filled by a free ILM flap. Therefore, a free ILM flap does not support the healing process but appears as a patch inserted into the FTMH without a restoration of the intraretinal layers and subsequently, with no functional improvement. A free ILM flap must work as a scaffold and not as a patch.

The strength of this technique is in its dual effect, which is caused by a surgically induced MD that promotes the approximation of FTMH edges by increasing the retinal elasticity and by synergistically accommodating the centripetal tractive forces given by the gliosis of Muller cells induced by the free ILM flap.

Nevertheless, cell re-proliferation is not the only mechanism determining foveal restoration. The crucial event is the contact between the edges of the FTMH favored by a combination of the two described surgical techniques. That prevents a communication between the macular hole and the vitreous chamber favoring its closure.

As mentioned, several surgical techniques have been proposed for the treatment of refractory FTMH [18,29,30,31]. However, the surgical approach proposed by the authors is not a new surgical technique. It is indeed the consequence of the combined surgical procedures described with the aim of enhancing surgical efficacy. The comparison with other alternative surgical techniques focuses essentially on the choice of anatomical tissue to be used as a flap compared to the ILM. In the literature, surgical techniques associated with the use of amniotic membrane, autologous retinal patch have been reported on [18,29,30,31]. We believe that the use of amniotic membrane could be a valid alternative, in the absence of ILM available during surgery. This offers the advantage of being able to create a patch of the correct size before inserting it into the eye and to avoid peeling maneuvers that are sometimes difficult, such as finding the ILM tissue to create an autologous patch. However, we do not believe that creating a retinal patch has a great advantage as the surgical maneuver is invasive and does not bring any additional advantages compared to the ILM or the amniotic membrane.

An important aspect that the authors want to underline is the timing of surgery. From our experience and the results obtained, it emerges that functional recovery is better the earlier the surgical treatment takes place.

The current study had a few limitations: as it is a case series, the number of patients is limited to those of our cohort and its results may need validation via a larger series or a clinical trial. Furthermore, the combined technique that we described was not matched with a control group, as the authors routinely perform it in cases of refractory FTMH. Finally, the authors overlooked on purpose the trend of BCVA over time, but considered as a functional parameter the BCVA 6 months post-surgery. We made that choice as BVCA at the 6 month timeframe is regarded as stable and not further influenced by the morphological and anatomical changes of the fovea that occur in the first months post-surgery. The aim of this study was to evaluate the percentage of anatomical and functional success of the hereby described combined surgery, and to evaluate whether there are pre-operative prognostic factors and post-operative morphological markers related to functional recovery.

To summarize, the key points of the proposed surgical technique are the following:

(1) it combines and enhances the effect of two well-known surgical procedures by adding them together synergically;
(2) it offers a new approach to the treatment of refractory FTMH, which would be useful in triggering retinal healing towards the complete restoration of the foveal tissue.

Even though further studies need to be performed to confirm the aforementioned hypotheses and the results obtained in our cohort, the authors suggest that this approach may be beneficial both anatomically and functionally in eyes affected by refractory FTMH.

## Figures and Tables

**Table 1:** Demographic, functional, pre and postoperative data.

Demographic Parameters of Patients (14 Eyes of 14 Patients)		
Age years	mean ± [range]	69.2 ± 4.8 [59–79]
Gender female/male	number (%)	6/8 (42.9/57.1)
Eye right/left	number (%)	6/8 (42.9/57.1)
Pre-operative Clinical Data		
Axial length (AL) Millimeters (mm)	mean ± [range]	24.6 ± 1.2 [22.9–26.3]
Best corrected visual acuity (BCVA) (logarithm of minimum angle of resolution)	mean ± [range]	1.1 ± 0.1 [1.4–1]
Pre-operative Tomographic Data of FTMH		
Base diameter μm	mean ± [range]	760.2 ± 219.8 [368–1120]
Minimum diameter μm	mean ± [range]	316.9 ± 126.4 [134–450]
Morphology of FTMH cuff/flat pattern	number (%)	7/7 (50/50) [134–450]
Time of surgery	mean ± [range]	2.3 ± 1.8 [1–6]
Post-operative BCVA (6th month) (logarithm of minimum angle of resolution)	mean ± [range]	0.48 ± 0.3 [1.1–0.3]
Post-operative Morphological Parameters (6th month of follow-up)		
FTMH closure rate yes-no	number (%)	14/0 (100/0)
Foveal profile Regular/flat/inverted	number (%)	7/5/2 (50/35/14.3)
Intact outer retinal layers yes-no	number (%)	12/2 (85.7/14.3)

## Data Availability

The raw data are stored at the Ophthalmology Unit, Surgery Department, Guglielmo da Saliceto hospital, Piacenza Italy, and are available upon request.

## Supplementary Materials

The following supporting information can be downloaded at: https://www.mdpi.com/article/10.3390/jcm14062123/s1, Video S1: surgical technique.
